# Significance of Blood Transfusion Units in Determining the Probability of Mortality among Elderly Trauma Patients Based on the Geriatric Trauma Outcome Scoring System: A Cross-Sectional Analysis Based on Trauma Registered Data

**DOI:** 10.3390/ijerph15102285

**Published:** 2018-10-18

**Authors:** Shao-Chun Wu, Cheng-Shyuan Rau, Pao-Jen Kuo, Hang-Tsung Liu, Shiun-Yuan Hsu, Ching-Hua Hsieh

**Affiliations:** 1 Department of Anesthesiology, Kaohsiung Chang Gung Memorial Hospital, Chang Gung University and College of Medicine, Kaohsiung 83301, Taiwan; shaochunwu@gmail.com; 2 Department of Neurosurgery, Kaohsiung Chang Gung Memorial Hospital, Chang Gung University and College of Medicine, Kaohsiung 83301, Taiwan; ersh2127@cloud.cgmh.org.tw; 3 Department of Plastic Surgery, Kaohsiung Chang Gung Memorial Hospital, Chang Gung University and College of Medicine, Kaohsiung 83301, Taiwan; bow110470@gmail.com; 4 Department of Trauma Surgery, Kaohsiung Chang Gung Memorial Hospital, Chang Gung University and College of Medicine, Kaohsiung 83301, Taiwan; htl1688@yahoo.com.tw (H.-T.L.); ah.lucy@hotmail.com (S.-Y.H.)

**Keywords:** mortality, blood transfusion, packed red blood cells, Geriatric Trauma Outcome Score, the Trauma and Injury Severity Score

## Abstract

*Background*: For elderly trauma patients, a prognostic tool called the Geriatric Trauma Outcome Score (GTOS), where GTOS = (age) + (ISS × 2.5) + (22 if any packed red blood cells (pRBCs) were transfused within 24 h after admission), was developed for predicting mortality. In such calculation, a score of 22 was added in the calculation of GTOS regardless of the transfused units of blood. This study aimed to assess the effect of transfused blood units on the mortality outcomes of the elderly trauma patients who received blood transfusion (BT). *Methods*: Detailed data of 687 elderly trauma patients aged ≥65 years who were transfused with pRBCs within 24 h after admission into a level I trauma center between 1 January 2009 and 31 December 2016 were retrieved from the Trauma Registry System database. Based on the units of pRBCs transfused, the study population was divided into two groups to compare the mortality outcomes between these groups. Adjusted odds ratios (AORs) with its 95% confidence intervals (CIs) for mortality were calculated by adjusting sex, pre-existing comorbidities, and GTOS. *Results*: When the cut-off value of BT was set as 3 U of pRBCs, patients who received BT ≥ 3 U had higher odds of mortality than those who received BT < 3 U (OR, 3.0; 95% CI, 1.94–4.56; *p* < 0.001). Patients who received more units of pRBCs still showed higher odds of mortality than their counterparts. After adjusting for sex, pre-existing comorbidities, and GTOS, comparison revealed that the patients who received BT of 3 U to 6 U had a 1.7-fold adjusted odds of mortality than their counterparts. The patients who received BT ≥ 8 U and 10 U had a 2.1-fold (AOR, 2.1; 95% CI, 1.09–3.96; *p* < 0.001) and 4.4-fold (AOR, 4.4; 95% CI, 2.04–9.48; *p* < 0.001) adjusted odds of mortality than those who received BT < 8 U and <10 U, respectively. *Conclusions*: This study revealed that the units of BT did matter in determining the probability of mortality. For those who received more units of blood, the mortality may be underestimated according to the GTOS.

## 1. Background

The progressive aging of the populations has led to a significant increase in the number of elderly patients who sustained trauma. Compared with younger adults, the elderly aged 65 years and older have a higher mortality after trauma [1]. While the total trauma population has a mortality rate of 12%, trauma accounts for 28% of mortality in geriatric patients [2]. With an equivalent injury burden, the elderly patients have a higher long-term risk of mortality after trauma than their younger counterparts [3,4].

To predict the mortality of trauma patient in order to help make complex decisions and potentially assist in determining the futility of care, the Trauma and Injury Severity Score (TRISS) was developed to estimate the survival probability of an individual patient with trauma based on logistic regression analysis of variables, including age, anatomical variable (Injury Severity Score; ISS), physiological variable (revised trauma score), and different coefficients for blunt and penetrating injuries [5]. However, TRISS was not designed and validated specifically for the elderly population, but for young patients with high-energy trauma [6]. It also requires time and adjustment by coefficients that are not updated or adapted to the different geographical areas in which it is used [6].

In recent years, a new useful scoring system for the elderly population called the Geriatric Trauma Outcome Score (GTOS) was developed. It was derived through analysis of roughly 3841 patients from a single center using logistic regression [7] and presented using the following formula: GTOS = age + (ISS × 2.5) + 22 (if pRBCs were transfused within 24 h) [7,8]. To assist with early goal-setting conversations after injury in the elderly, this scoring system is simpler and easier to use within 24 h after admission [7]. The selected GTOS scores and their related probabilities of dying were as follows: 205 = 75%, 233 = 90%, 252 = 95%, and 310 = 99% [7]. In a multicenter external validation study, the GTOS can estimate the probability of dying of 18,282 patients with a high degree of accuracy with area under the curve (AUC) being 0.86, in comparable with the AUC (0.82) of the original Parkland sample of 3841 patients [8]. In addition, the GTOS and TRISS function similarly and accurately in predicting the probability of death for injured elders in a multicenter sample [9].

GTOS has the advantages of a single formula, fewer variables, and no reliance on data collected in the emergency room or by other observers [9]. However, the accuracy of GTOS in predicting the mortality of trauma patients remained controversial. It had been reported that the accuracy of GTOS in predicting in-hospital survival was lower than that of TRISS [10]. In addition, with a misclassification rate of 17.6% and Brier score of 0.13, the GTOS is not adept at predicting 1-year mortality [11]. This result does not support the use of GTOS in place of the TRISS in predicting the mortality of elderly trauma patients [11]. Notably, blood transfusion (BT) is reported to be associated with increased morbidity and mortality [12], and massive blood transfusion is associated with a variety of complications such as coagulation abnormalities, immunosuppression, hypothermia, lung injury, and infection [13]. In the calculation of GTOS, a score of 22 is added if packed red blood cells (pRBCs) were transfused within 24 h. However, adding the score of 22 into the GTOS in all trauma elderly patients regardless of the blood unit transfused may be too simplified to estimate the mortality outcome. Therefore, this study aimed to assess the effect of transfused blood units on the mortality outcomes of the trauma elderly patients who received BT after adjusting the baseline characteristic including sex, pre-existing comorbidities, and GTOS.

## 2. Methods

### 2.1. Ethics Statement

The institutional review board of the Kaohsiung Chang Gung Memorial Hospital, a level I regional trauma center in southern Taiwan [14,15], approved this study (reference number: 201800434B0). The need for informed consent was waived because of the retrospective nature of the study using the registered data.

### 2.2. Study Population

This study reviewed 27,462 patients who sustained a trauma injury and admitted in the hospital from 1 January 2009 to 31 December 2016. Among 7068 elderly patients aged ≥65 years, this study only included 687 patients who were transfused with pRBCs within 24 h after arrival at the emergency department (ED). The following patient information were retrieved: age; sex; comorbidities, such as diabetes mellitus (DM), hypertension (HTN), coronary artery disease, congestive heart failure, cerebral vascular accident, and end-stage renal disease (ESRD); ISS; units of pRBCs transfused within 24 h; and mortality in the hospital. GTOS was specified using the following formula: GTOS = age + (ISS × 2.5) + 22 (if pRBCs were transfused within 24 h after admission).

### 2.3. Statistical Analysis

Statistical analysis was performed using the Statistical Package for Social Science software, version 22 (IBM Corp., Armonk, NY, USA). For the continuous variables, Levene’s test was used to estimate the homogeneity of variance; the one-way analysis of variance with Games–Howell post-hoc test was used to evaluate the differences among patient groups. Continuous data were expressed as mean ± standard deviation. The ISS was expressed as median and interquartile range (IQR, Q1–Q3). The odds ratios (ORs) with 95% CIs of the associated conditions of the patients were presented. Mortality of patients in the hospital was the primary outcome of the study. Based on the units of pRBCs transfused, the study population was divided into two groups to compare the outcome between these groups. Adjusted ORs (AORs) with the 95% CIs for mortality, adjusted by sex, pre-existing comorbidities, and GTOS or adjusted by age, sex, pre-existing comorbidities, ISS, and GTOS were calculated. The units of pRBCs transfused within 24 h were evaluated to determine the cut-off points that could predict the risk of mortality among these elderly patients by plotting specific receiver operating characteristic (ROC) curves. The accuracy of parameter in predicting the mortality outcomes was calculated based on the maximal Youden index, calculated as sensitivity + specificity – 1, to reflect the maximal correct classification accuracy. A two-tailed *p* value < 0.05 was considered significant for all analyses.

## 3. Results

### 3.1. ROC Curve Analysis

**Figure 1:**
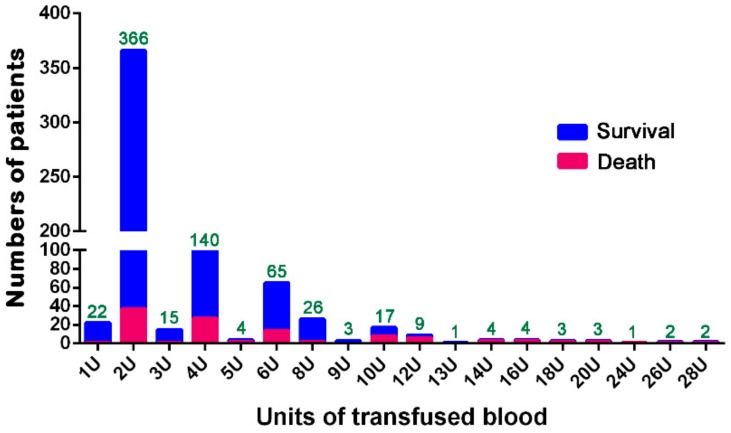
Numbers of patients receiving blood transfusion with various units of pRBCs within 24 h after arrival at the emergency room.

**Figure 2:**
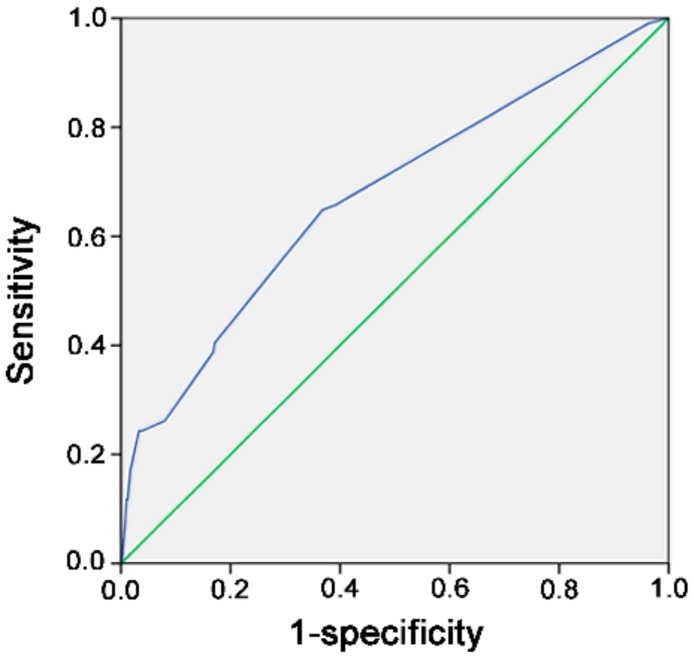
Receiver operating characteristic curve analysis to identify cut-off values for predicting mortality based on the units of transfused pRBCs.

As shown in Figure 1, among the 687 patients studied, majority of them were transfused with 2 U of pRBCs (*n* = 366, 53.2%), followed by those who received 4 U of pRBCs (*n* = 140, 20.4%) and those who received 6 U of pRBCs (*n* = 65, 9.5%). Forty-six (6.7%) patients received ≥10 U of pRBCs. To predict the probability of mortality among elderly patients, the cut-off values of pRBCs transfused within 24 h were evaluated by ROC curve analysis. The maximal Youden index, calculated as sensitivity + specificity – 1, was calculated to reflect the maximal correct classification accuracy. According to the ROC curve analysis (Figure 2), a BT of 3.5 U of pRBCs was identified as the best cut-off value for predicting mortality outcomes, with AUCs of 0.673. Prediction of mortality based solely on the units of transfused pRBCs is not good.

### 3.2. Characteristics and Outcomes of Patients

The study population was divided into two groups to compare the outcome according to the units of pRBCs transfused. First, with the cut-off value of BT set as 2 U of pRBCs (Table 1), a total of 687 patients were divided into two groups: those who received pRBCs equal or more than 2 units (BT ≥ 2 U, *n* = 665) and those who received pRBCs < 2 U (BT < 2 U, *n* = 22). In terms of age or sex, no significant difference was observed between the patients who received BT of ≥2 U and those who received BT of 2 U. The prevalence rates of comorbidities among individuals were not significantly different between the patients who received BT ≥ 2 U and those who received BT < 2 U. By contrast, the prevalence rates of ESRD were significantly lower in the patients who received BT ≥ 2 U than in those who received BT < 2 U. No significant difference in ISS, GTOS, and odds of mortality was observed between patients who received BT ≥ 2 U and those who received BT < 2 U.

When the cut-off value of BT was set as 3 U of pRBCs (Table 2), no significant difference was observed between patients who received BT ≥ 3 U and those who received BT < 3 U in terms of age. By contrast, patients who received BT ≥ 3 U had a significant male predominance and a lower prevalence of pre-existing DM and HTN compared with those who received BT < 3 U. The ISS and GTOS of the patients who received BT ≥ 3 U were significantly higher than those who received BT < 3 U.

The patients who received BT ≥ 3 U had a higher odds of mortality than those who received BT < 3 U (OR, 3.0; 95% CI, 1.94–4.56; *p* < 0.001). When the cut-off value of BT was set as 4 U of pRBCs (Table 3), the outcomes of patients who received BT ≥ 4 U and those who received BT < 4 U were similar to the patient outcomes when the cut-off value of BT was set as 3 units. The patients who received BT ≥ 4 U were predominantly men, had lower prevalence of pre-existing DM and HTN, and had higher ISS and GTOS than those who received BT < 4 U. The patients who received BT ≥ 4 U had a similar higher odds of mortality compared with those who received BT < 4 U (OR, 3.2; 95% CI, 2.07–4.85; *p* < 0.001).

When the cut-off value of BT was set as 6 units of pRBCs (Table 4), results demonstrated that the patients who received BT ≥ 6 U had a younger age, were predominantly men, and had lower prevalence of pre-existing HTN than those who received BT < 6 U. The ISS and GTOS of the patients who received BT ≥ 6 U were significantly higher than those who received BT < 6 U. The patients who received BT ≥ 6 U had a higher odds of mortality than those who received BT < 6 U (OR, 3.1; 95% CI, 2.01–4.85; *p* < 0.001). When the cut-off value of BT was set as 8 units of pRBCs (Table 5), the outcomes between the patients who received BT ≥ 8 U and those who received BT < 8 U were similar to the patient outcomes when the cut-off value of BT was set as 6 units. By contrast, the patients who received BT ≥ 8 U had a 4.1-fold risk of mortality than those who received BT < 8 U (OR, 4.1; 95% CI, 2.42–6.85; *p* < 0.001).

When the cut-off value of BT was set as 10 U of pRBCs (Table 6), results demonstrated that the patients who received BT ≥ 10 U had younger age and lower prevalence of pre-existing HTN than those who received BT < 10 U, while no significant difference was observed between the patients who received BT ≥ 10 U and those who received BT < 10 U in terms of sex. The ISS and GTOS of the patients who received BT ≥ 10 U were significantly higher than those who received BT < 10 U. The patients who received BT ≥ 10 U had a much higher odds of mortality than those who received BT < 10 U (OR, 9.4; 95% CI, 5.02–17.70; *p* < 0.001).

### 3.3. Adjusted Mortality Outcomes of the Patients

**Figure 3:**
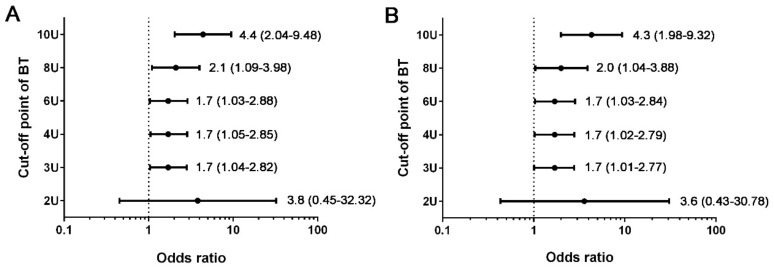
Adjusted odds of mortality between groups of comparative patients with various cut-off points of BT adjusted by sex, pre-existing comorbidities, and GTOS (**A**) or adjusted by sex, pre-existing comorbidities, GTOS, and additional age and ISS (**B**).

To attenuate the confounding effect of the baseline patient characteristics on the assessment of the mortality outcomes, the adjusted odds of mortality between groups of comparative patients with various cut-off points of BT were calculated based on two scenarios: one is adjusted by sex, pre-existing comorbidities, and GTOS (Figure 3A), and the other one is adjusted by sex, pre-existing comorbidities, GTOS, and additional age and ISS (Figure 3B). In the condition of adjustment by sex, pre-existing comorbidities, and GTOS, the patients who received BT ≥ 3 U, 4 U, and 6 U all had a 1.7-fold adjusted odds of mortality compared with those who received BT < 3 U, 4 U, and 6 U, respectively. The patients who received BT ≥ 8 U had a 2.1-fold adjusted odds of mortality (AOR, 2.1; 95% CI, 1.09–3.96; *p* < 0.001) compared with those who received BT < 8 U. The patients who received BT ≥ 10 U had a 4.4-fold adjusted odds of mortality (AOR, 4.4; 95% CI, 2.04–9.48; *p* < 0.001) compared with those who received BT < 10 U. As shown in Figure 3B, with age and ISS as the additional variables for adjustment, the adjusted mortality outcomes were similar to those adjusted by sex, pre-existing comorbidities, and GTOS, albeit the adjustment of baseline conditions under the former scenario is stricter than that in the latter scenario, owing to the fact that the variables of age and ISS had already been included in the calculation of GTOS. This result indicated the conclusion, that the unit of the units of BT did matter in determining the probability of mortality, remained the same regardless of the variables chosen for adjustment in both two conditions.

## 4. Discussion

This study assessed the effect of transfused blood units on the mortality outcomes of the elderly trauma patients by adjusting sex, pre-existing comorbidities, and GTOS and revealed that the units of BT did matter in determining the probability of mortality. This means, if the transfused units of blood were different, the same GTOS of patients in similar baseline characteristics would be associated with different mortality outcome. Thus, the probability of mortality would be underestimated, especially in the patients transfused with large amount of blood. When the cut-off value of BT was set as 3 U of pRBCs, patients receiving BT ≥ 3 U had higher odds of mortality than those receiving BT < 3 U (OR, 3.0; 95% CI, 1.94–4.56; *p* < 0.001). Patients receiving more units of pRBCs had higher odds of mortality than their counterparts. Although the prediction of mortality based solely on the units of transfused pRBCs is not good (AUC, 0.673), the patients receiving 3 U to 6 U of BT had a 1.7-fold adjusted odds of mortality compared with their counterparts. The patients receiving BT ≥ 8 U and 10 U had a 2.1- and 4.4-fold adjusted odds of mortality compared with those receiving BT < 8 U and <10 U, respectively.

In a comparison of results of RTS or ISS, TRISS was a stronger predictor of mortality in elderly trauma patients as result of the combination of both anatomical and physiological parameters [16]. A hybrid model incorporating the anatomical and physiological aspects of the trauma patients is expected to have heightened discriminatory abilities for predicting mortality outcomes. TRISS can estimate the survival probability of an individual patient with trauma based on the following variables: age, ISS (anatomical variable), RTS (physiological variable), and various coefficients for blunt and penetrating injuries [5]. The GTOS uses the covariates of age, ISS, and BT [9]. Hemorrhagic shock is a leading cause of mortality within the trauma population. Although blood transfusion may indicate an important physiological response to a drop of systolic blood pressure or a risk associated with penetration injury, the use of BT as the sole physiological variable may not be accurate as that calculated from RTS, which is made up of three categories: Glasgow Coma Scale (GCS), systolic blood pressure, and respiratory rate [17]. A very low GCS score, a variable that is not used in the calculation of GTOS, is a strong clinical indicator of prognosis in patients with traumatic brain injury [18]. Patients with a GCS score below 12 was associated with a twofold increase in mortality rate (39% vs 83%) compared with those who had a GCS score equal or higher than 12 [18]. Furthermore, the units of blood transfused may indicate profound hemorrhage shock. In a systematic review of 45 studies including 272,596 patients, transfusions of pRBCs are associated with increased morbidity and mortality and presented as an independent risk factor for infection [12]. The pooled odds ratios for developing an infectious complication and acute respiratory distress syndrome were 1.8 (95% CI, 1.5–2.2) and 2.5 (95% CI, 1.6–3.3), respectively [12]. While the incidence of massive transfusion is relatively low, patients requiring massive transfusions have a high mortality [13] and are at risk of developing a variety of complications such as coagulopathy, immunosuppression, hypothermia, and lung injury [19]. The units of blood transfusion did matter in determining the probability of mortality calculated by the Geriatric Trauma Outcome Score in the trauma elderly. For those who received more units of blood, the mortality may be underestimated according to the GTOS.

We presumed that the GTOS formula offers a simple and straightforward method of communicating the risk of death to patients’ families and decision making. It is a tool that can be easily used in the clinical setting to assist with early goal-setting conversations with an elderly trauma patient. However, aside from the fact that the GTOS is only applicable to the patient's index admission [8], the score can only be used to predict the probability of mortality but not for estimating functional recovery [8] and the lack of preexisting conditions of the elderly [8]. Hence, our results reflect the need to adapt and update the predictive scales of the study population based on the different units of blood transfused as an effort to resuscitate a patient within 24 h. If the patient had received BT equal or more than 3 U, then a higher probability for mortality should be weighted on the patient against that predicated by the GTOS. If there is a massive blood transfusion (i.e., BT ≥ 10 U), then the mortality rate even would be four odds of mortality than that predicted by the GTOS. In recent years, the use of plasma and higher ratios of red blood cells to plasma for transfusions has been increasing [20]. The balanced resuscitation with plasma, platelets, and red blood cells in a 1:1:1 ratio minimizes coagulopathy and thus improves outcomes. Moreover, the use of this method became popular in many trauma centers [21]. Obviously, the calculation of GTOS with solely addition of score 22 if transfused pRBCs at 24 h would lead to some limitation when the component of blood or fluid transfused was not considered.

There were some other limitations in this study. First, the current study has a retrospective design. We can only assume that all patients had received a uniform management and resuscitation in the clinical setting. Second, the patients declared to be dead at the scene of the accident or upon arrival at the ED were not included in the Trauma Registry System, and this might have resulted in selection bias upon estimation of mortality outcome. Third, the indication for blood transfusion and the units of blood required varied among the physicians or surgeons in the ED and may result in the selection bias, particularly considering that the favor for a balanced resuscitation is quite different among the caring medical staffs. Fourth, this study was performed using the registered data of one trauma center and thus led to a limitation on its generalizability. Fifth, when the cut-off value of BT unit was set at 2 U, there were 22 patients had received BT less than 2 U in the control group. The patient number is relatively small for statistical analysis and may be underpowered to support the conclusion for this cut-off point of value. However, with the cut-off point of BT unit being higher, there would be more patients in the control group for the statistical analysis and it is noted that there is a tendency that the higher the cut-off point of BT unit, the higher the odds of mortality was found. Further, it should be considered to incorporate this model into a primary health care system for the patient [22].

## 5. Conclusions

This study assesses the effect of transfused blood units on the mortality outcomes of elderly trauma patients by adjusting sex, pre-existed comorbidities, and GTOS and revealed that the units of BT did matter in determining the probability of mortality. For those who received higher units of blood transfusion, the mortality may be underestimated according to the GTOS.

## Figures and Tables

**Table 1:** Comparison between patients receiving RBC transfusion of <2 U and those receiving RBC blood transfusion of ≥2 U.

Variables	BT ≥ 2 U *n* = 665		BT < 2 U *n* = 22		Odds Ratio(95% CI)		*p*
Age (years)	77.1	±7.5	79.2	±5.9	-		0.199
Gender, *n* (%)							0.665
Male	281	(42.3)	8	(36.4)	1.3	(0.53–3.09)	
Female	384	(57.7)	14	(63.6)	0.8	(0.32–1.89)	
Co-morbidities, *n* (%)							
DM	191	(28.7)	9	(40.9)	0.6	(0.25–1.38)	0.235
HTN	376	(56.5)	15	(68.2)	0.6	(0.24–1.51)	0.382
CAD	69	(10.4)	3	(13.6)	0.7	(0.21–2.54)	0.719
CHF	14	(2.1)	2	(9.1)	0.2	(0.05–1.01)	0.090
CVA	60	(9.0)	1	(4.5)	2.1	(0.28–15.76)	0.712
ESRD	19	(2.9)	3	(13.6)	0.2	(0.05–0.68)	0.030
ISS (median, IQR)	9	(9–20)	9	(9–13.8)	-		0.020
<16, *n* (%)	410	(61.7)	17	(77.3)	0.5	(0.17–1.30)	0.181
16–24, *n* (%)	113	(17.0)	3	(13.6)	1.3	(0.38–4.46)	0.783
>24, *n* (%)	142	(21.4)	2	(9.1)	2.7	(0.63–11.75)	0.194
GTOS	135.8	±21.9	131.0	±13.4	-		0.115
Mortality, *n* (%)	110	(16.5)	1	(4.5)	4.2	(0.55–31.27)	0.154

RBC = red blood cell; BT = blood transfusion; CAD = coronary artery disease; CHF = congestive heart failure; CI = confidence interval; CVA = cerebral vascular accident; DM = diabetes mellitus; ESRD = end-stage renal disease; GTOS = Geriatric Trauma Outcome Score; HTN = hypertension; IQR = interquartile range; ISS = injury severity score.

**Table 2:** Comparison between patients receiving RBC transfusion of <3 U and those receiving RBC transfusion of ≥3 U.

Variables	BT ≥ 3 U *n* = 299		BT < 3 U *n* = 388		Odds Ratio (95% CI)		*p*
Age (years)	76.8	±7.6	77.5	±7.4	-		0.235
Gender, *n* (%)							0.019
Male	141	(42.7)	148	(38.1)	1.4	(1.07–1.97)	
Female	158	(52.8)	240	(61.9)	0.7	(0.51–0.94)	
Co-morbidities, *n* (%)							
DM	75	(25.1)	125	(32.2)	0.7	(0.50–0.99)	0.043
HTN	151	(50.5)	240	(61.9)	0.6	(0.46–0.85)	0.003
CAD	33	(11.0)	39	(10.1)	1.1	(0.68–1.81)	0.707
CHF	5	(1.7)	11	(2.8)	0.6	(0.20–1.70)	0.446
CVA	21	(7.0)	40	(10.3)	0.7	(0.38–1.14)	0.139
ESRD	5	(1.7)	17	(4.4)	0.4	(0.14–1.02)	0.050
ISS (median, IQR)	16	(9–25)	9	(9–16)	-		<0.001
<16, *n* (%)	145	(48.5)	282	(72.7)	0.4	(0.26–0.49	<0.001
16–24, *n* (%)	57	(19.1)	59	(15.2)	1.3	(0.88–1.96)	0.184
>24, *n* (%)	97	(32.4)	47	(12.1)	3.5	(2.36–5.14)	<0.001
GTOS	142.1	±23.7	130.8	±18.7	-		<0.001
Mortality, *n* (%)	73	(24.4)	38	(9.8)	3.0	(1.94–4.56)	<0.001

BT = blood transfusion; CAD = coronary artery disease; CHF = congestive heart failure; CI = confidence interval; CVA = cerebral vascular accident; DM = diabetes mellitus; ESRD = end-stage renal disease; GTOS = Geriatric Trauma Outcome Score; HTN = hypertension; IQR = interquartile range; ISS = injury severity score.

**Table 3:** Comparison between patients receiving RBC transfusion of <4 U and those receiving RBC transfusion of ≥4 U.

Variables	BT ≥ 4 U *n* = 284		BT < 4 U *n* = 403		Odds Ratio (95% CI)		*p*
Age (years)	76.6	±7.5	77.5	±7.5	-		0.118
Gender, *n* (%)							0.010
Male	136	(47.9)	153	(38.0)	1.5	(1.10–2.04)	
Female	148	(52.1)	250	(62.0)	0.7	(0.49–0.91)	
Co-morbidities, *n* (%)							
DM	69	(24.3)	131	(32.5)	0.7	(0.47–0.94)	0.021
HTN	141	(49.6)	250	(62.0)	0.6	(0.44–0.82)	0.001
CAD	31	(10.9)	41	(10.2)	1.1	(0.66–1.77)	0.801
CHF	5	(1.8)	11	(2.7)	0.6	(0.22–1.86)	0.454
CVA	21	(7.4)	40	(9.9)	0.7	(0.42–1.26)	0.278
ESRD	5	(1.8)	17	(4.2)	0.4	(0.15–1.12)	0.081
ISS (median, IQR)	16	(9–25)	9	(9–16)	-		<0.001
<16, *n* (%)	132	(46.5)	295	(73.2)	0.3	(0.23–0.44)	<0.001
16–24, *n* (%)	55	(19.4)	61	(15.1)	1.3	(0.90–2.01)	0.149
>24, *n* (%)	97	(34.2)	47	(11.7)	3.9	(2.66–5.81)	<0.001
GTOS	142.9	±23.9	130.6	±18.5	-		<0.001
Mortality, *n* (%)	72	(25.4)	39	(9.7)	3.2	(2.07–4.85)	<0.001

BT = blood transfusion; CAD = coronary artery disease; CHF = congestive heart failure; CI = confidence interval; CVA = cerebral vascular accident; DM = diabetes mellitus; ESRD = end-stage renal disease; GTOS=Geriatric Trauma Outcome Score; HTN = hypertension; IQR = interquartile range; ISS = injury severity score.

**Table 4:** Comparison between patients receiving RBC transfusion of <6 U and those receiving RBC transfusion of ≥6 U.

Variables	BT ≥ 6 U *n* = 140		BT < 6 U *n* = 574		Odds Ratio (95% CI)		*p*
Age (years)	75.7	±6.9	77.6	±7.6	-		0.008
Gender, *n* (%)							0.001
Male	77	(55.0)	212	(38.8)	1.9	(1.33–2.81)	
Female	63	(45.0)	335	(61.2)	0.5	(0.36–0.75)	
Co-morbidities, *n* (%)							
DM	33	(23.6)	167	(30.5)	0.7	(0.46–1.08)	0.118
HTN	65	(46.4)	326	(59.6)	0.6	(0.40–0.85)	0.006
CAD	20	(14.3)	52	(9.5)	1.6	(0.91–2.76)	0.121
CHF	3	(2.1)	13	(2.4)	0.9	(0.25–3.20)	1.000
CVA	5	(3.6)	56	(10.2)	0.3	(0.13–0.83)	0.019
ESRD	4	(2.9)	18	(3.3)	0.9	(0.29–2.60)	1.000
ISS (median, IQR)	18.5	(9–27)	9	(9–16)	-		<0.001
<16, *n* (%)	51	(36.4)	376	(68.7)	0.3	(0.18–00.38)	<0.001
16-24, *n* (%)	31	(22.1)	85	(15.5)	1.5	(0.98–2.45)	0.076
>24, *n* (%)	58	(41.4)	86	(15.7)	3.8	(2.52–5.70)	<0.001
GTOS	147.2	±25.2	132.7	±19.7	-		<0.001
Mortality, *n* (%)	43	(30.7)	68	(12.4)	3.1	(2.01–4.85)	<0.001

BT = blood transfusion; CAD = coronary artery disease; CHF = congestive heart failure; CI = confidence interval; CVA = cerebral vascular accident; DM = diabetes mellitus; ESRD = end-stage renal disease; GTOS = Geriatric Trauma Outcome Score; HTN = hypertension; IQR = interquartile range; ISS = injury severity score.

**Table 5:** Comparison between patients receiving RBC transfusion of <8 U and those receiving RBC transfusion of ≥8 U.

Variables	BT ≥ 8 U *n* = 75		BT < 8 U *n* = 612		Odds Ratio (95% CI)		*p*
Age (years)	75.0	±6.7	77.4	±7.5	-		0.008
Gender, *n* (%)							0.025
Male	41	(54.7)	248	(40.5)	1.8	(1.09–2.87)	
Female	34	(45.3)	364	(59.5)	0.6	(0.35–0.92)	
Co-morbidities, *n* (%)							
DM	17	(22.7)	183	(29.9)	0.7	(0.39–1.21)	0.226
HTN	29	(38.7)	362	(59.2)	0.4	(0.27–0.71)	0.001
CAD	11	(14.7)	61	(10.0)	1.6	(0.78–3.10)	0.229
CHF	2	(2.7)	14	(2.3)	1.2	(0.26–5.25)	1.000
CVA	2	(2.7)	59	(9.6)	0.3	(0.07–1.07)	0.050
ESRD	0	(0.0)	22	(3.6)	-	0.156	1.000
ISS (median, IQR)	21	(10–29)	9	(9–17)	-		<0.001
<16, *n* (%)	25	(33.3)	402	(65.7)	0.3	(0.16–0.43)	<0.001
16–24, *n* (%)	15	(20.0)	101	(16.5)	1.3	(0.69–2.32)	0.513
>24, *n* (%)	35	(46.7)	109	(17.8)	4.0	(2.45–6.65)	<0.001
GTOS	150.4	±27.1	133.9	±20.3	-		<0.001
Mortality, *n* (%)	29	(38.7)	82	(13.4)	4.1	(2.42–6.85)	<0.001

BT = blood transfusion; CAD = coronary artery disease; CHF = congestive heart failure; CI = confidence interval; CVA = cerebral vascular accident; DM = diabetes mellitus; ESRD = end-stage renal disease; GTOS = Geriatric Trauma Outcome Score; HTN = hypertension; IQR = interquartile range; ISS = injury severity score.

**Table 6:** Comparison between patients receiving RBC transfusion of <10 U and those receiving RBC transfusion of ≥10 U.

Variables	BT ≥ 10 U *n* = 46		BT < 10 U *n* = 641		Odds Ratio (95% CI)		*p*
Age (years)	75.0	±6.3	77.3	±7.5	-		0.045
Gender, *n* (%)							0.442
Male	22	(47.8)	267	(41.7)	1.3	(0.71–2.34)	
Female	24	(52.2)	374	(58.3)	0.8	(0.43–1.42)	
Co-morbidities, *n* (%)							
DM	11	(23.9)	189	(29.5)	0.8	(0.37–1.51)	0.503
HTN	14	(30.4)	377	(58.8)	0.3	(0.16–0.59)	<0.001
CAD	8	(17.4)	64	(10.0)	1.9	(0.85–4.25)	0.131
CHF	0	(0.0)	16	(2.5)	-	0.411	1.000
CVA	1	(2.2)	60	(9.4)	0.2	(0.03–1.59)	0.111
ESRD	0	(0.0)	22	(3.4)	-	0.390	1.000
ISS (median, IQR)	25	(16–32)	9	(9–17)	-		<0.001
<16, *n* (%)	10	(21.7)	417	(65.1)	0.1	(0.07–0.31)	<0.001
16–24, *n* (%)	8	(17.4)	108	(16.8)	1.0	(0.47–2.29)	1.000
>24, *n* (%)	28	(60.9)	116	(18.1)	7.0	(3.77–13.16)	<0.001
GTOS	158.2	±26.4	134.1	±20.5	-		<0.001
Mortality, *n* (%)	27	(58.7)	84	(13.1)	9.4	(5.02–17.70)	<0.001

BT = blood transfusion; CAD = coronary artery disease; CHF = congestive heart failure; CI = confidence interval; CVA = cerebral vascular accident; DM = diabetes mellitus; ESRD = end-stage renal disease; GTOS = Geriatric Trauma Outcome Score; HTN = hypertension; IQR = interquartile range; ISS = injury severity score.
